# Supplementary material for: Optimizing dsRNA sequences for RNAi in pest control and research with the dsRIP web platform
Source: BMC Biol. 2025 Apr 28;23:114. doi: 10.1186/s12915-025-02219-6 (PMC12039203; doi:10.1186/s12915-025-02219-6)
Supplement: Supplementary file 3 — Additional file 3: Supplementary figures and tables. Contains supplementary Table 1 and supplementary Figs. 1–4. Fig. S1 Other siRNAs tested in the Tribolium castaneum. Table S1 Scores and hazard ratios of dsRNA tested in Tribolium castaneum. Fig. S2 Length distribution in RISC-bound sRNA mapping to the respective dsRNA sequence. Fig. S3 Gene expression of Tc-gawky following single siRNA inserted or long complementary dsRNA injection. Fig. S4 Reproducibility of RISC-bound sRNA-seq experiments. [file 12915_2025_2219_MOESM3_ESM.docx]

**Additional File 3**


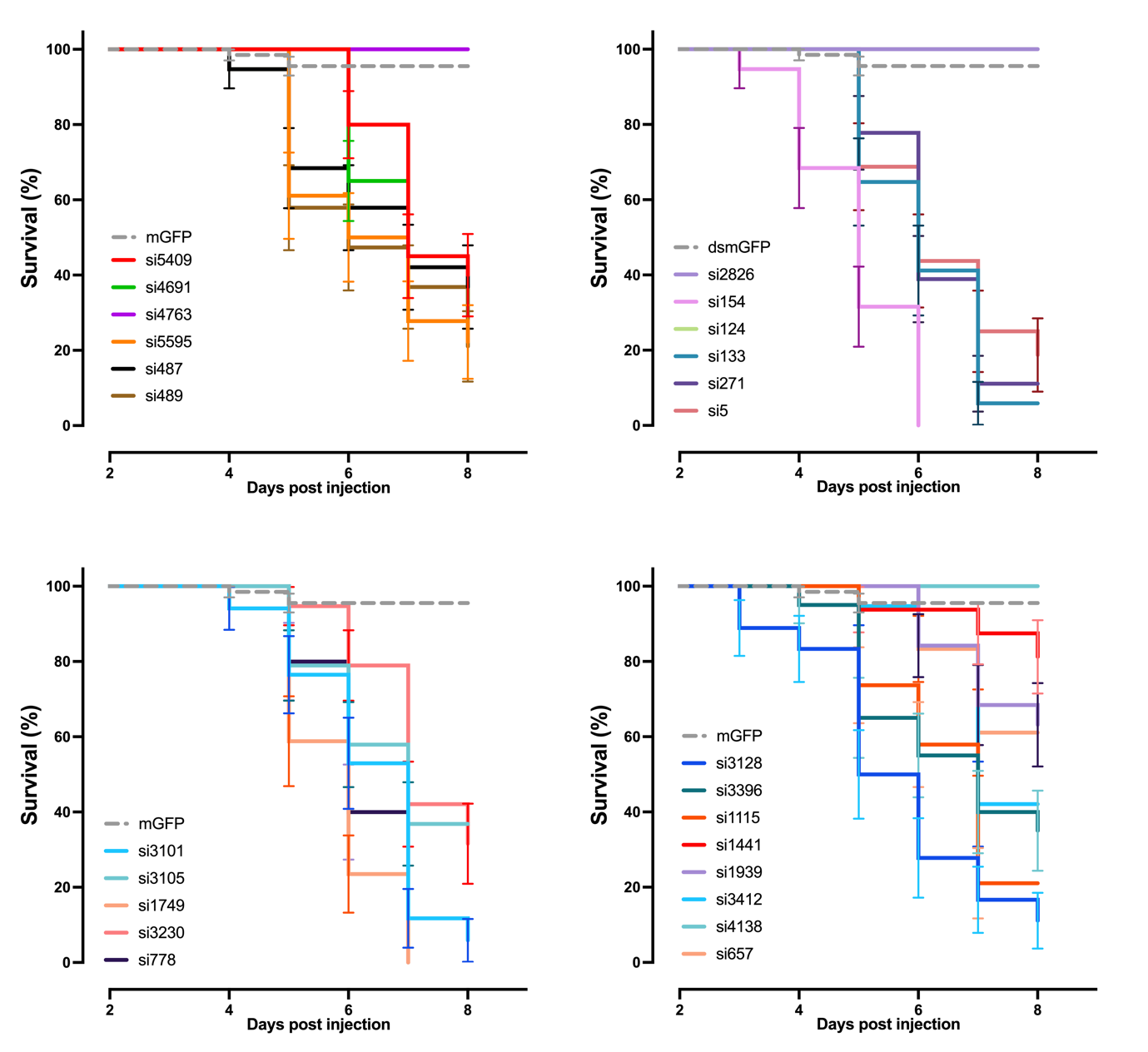


**Figure S1. Other siRNAs tested in the *Tribolium castaneum.*** L5 larvae (n = 20 per group) were injected with 1000 ng of dsRNA containing one siRNA, which was complementary to the essential gene *Tc-gawky*. siRNAs were named after the position they are targeting in the *Tc-gawky* mRNA. The Kaplan-Meier survival curves (mean survival ± standard error) were plotted for each siRNA along with the non-siRNA inserted dsmGFP as a control (same data in all plots).

**Table S1. Scores and hazard ratios of dsRNA tested in *Tribolium castaneum***

| Target gene | siRNA   score | Accessibility  score | Region | Hazard  ratio |
| --- | --- | --- | --- | --- |
| *Tc-gawky* | 74.3 | 73.8 | ORF | 36.8 |
| *Tc-gawky* | 66.4 | 67.4 | ORF | 5.75 |
| *Tc-gawky* | 71.4 | 85.4 | ORF | 23.2 |
| *Tc-gawky* | 78.4 | 78.5 | UTR | 9.78 |
| *Tc-klp61F* | 76.9 | 78 | ORF | 12.1 |
| *Tc-klp61F* | 69.7 | 70 | ORF | 4.88 |
| *Tc-klp61F* | 73.3 | 86.4 | ORF | 9.9 |
| *Tc-klp61F* | 77.8 | 78.7 | UTR | 10.7 |
| *Tc-nito* | 77 | 71.6 | ORF | 29.4 |
| *Tc-nito* | 65.3 | 73.1 | ORF | 3.0 |
| *Tc-nito* | 74.8 | 78.9 | UTR | 6.34 |
| *Tc-nito* | 78.2 | 74.6 | UTR | 12.4 |
| *Tc-hr3* | 75.1 | 75.1 | ORF | 11.1 |
| *Tc-hr3* | 72 | 72.1 | ORF | 1.55 |
| *Tc-hr3* | 76.3 | 80.4 | UTR | 5.17 |
| *Tc-hr3* | 77.8 | 76.6 | UTR | 11.8 |
| *Tc-eIF3a* | 75.3 | 66 | ORF | 6.5 |
| *Tc-eIF3a* | 65.1 | 58.1 | ORF | 4.14 |
| *Tc-eIF3a* | 73.4 | 80.5 | ORF | 5.39 |
| *Tc-rpt1* | 74.3 | 74.7 | ORF | 5.29 |
| *Tc-rpt1* | 67.6 | 57.6 | ORF | 4.45 |
| *Tc-rpt1* | 72.8 | 78.3 | ORF | 5.75 |
| *Tc-rpn7* | 76.1 | 79.2 | ORF | 10.4 |
| *Tc-rpn7* | 71 | 76.6 | ORF | 8.02 |
| *Tc-rpn7* | 76.2 | 76.4 | UTR | 6.14 |
| *Tc-cyp4g15* | 73.57 | 73.96 | ORF | 12.7 |
| *Tc-cyp4g15* | 69.47 | 69.5 | ORF | 11.9 |
| *Tc-cyp4g15* | 71.6 | 78.1 | ORF | 12.6 |


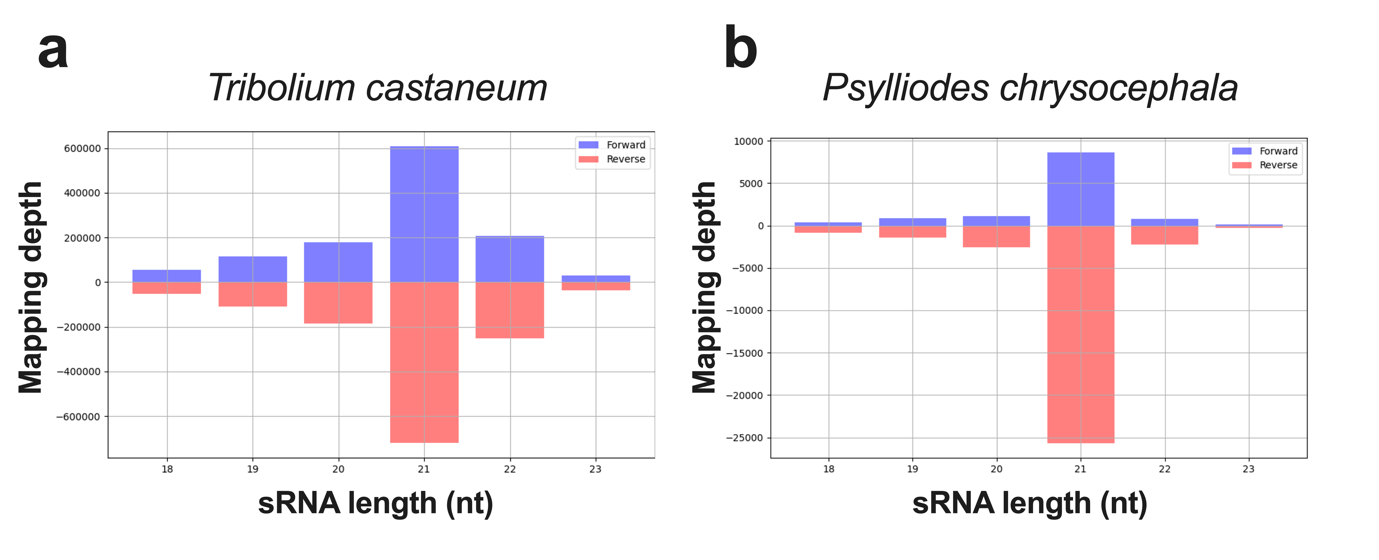


**Figure S2. Length distribution in RISC-bound sRNA mapping to the respective dsRNA sequence.** a) All RISC-bound sRNA-seq experiments in *T. castaneum* larvae were combined to plot the overall length distribution (n = 16). b) The RISC-bound sRNA length distribution in *P. chrysocephala* adults fed with dsmGFP (n = 1).

**Gene knockdown efficacy**

In Fig. 1 and Fig. 2, we focused on the impact of either single siRNA-inserted or fully complementary dsRNAs on the survival of *T. castaneum* larvae. Here, we measured the effect of selected siRNA-inserted dsRNAs and fully complementary dsRNAs targeting *Tc-gawky* on gene expression (Fig. S3). Among the single siRNA-inserted dsRNAs, si2443 and si3129 exhibited the highest knockdown effects (~60% reduction, P < 0.05) in gene expression relative to the dsmGFP control, consistent with their strong impact on survival (100% mortality within 8 days, see Fig. 1 in the main manuscript). In contrast, si1565 and si808 had significantly lower knockdown effects compared to si2443 and si3129, consistent with the observation that si1565 and si808 had little to no effects on survival (0%-40% mortality within 8 days, Fig. 1b).

Among the fully complementary dsRNAs targeting *Tc-gawky*, the one with a high siRNA score within the ORF had the most pronounced knockdown effect (~80% reduction, P < 0.05, Fig. S2), whereas the one with a low siRNA score within the ORF had the least knockdown effect (~30% reduction, P < 0.05). These findings were consistent with their respective impacts on survival (Fig. 2a). The other two dsRNAs showed intermediate effects on gene expression (50%-60% reduction, P < 0.05), in line with their effects on survival (Fig. 2a).

Interestingly, even dsRNAs with minimal impact on survival in Fig. 1 (e.g., si808 treatment resulted in 0% non-technical mortality, Fig. 1) still induced significant gene knockdown. However, since we only assessed gene expression at a single time point (3 days post-dsRNA treatment), we lack insights into potential dynamic variations in gene expression effects over time. These temporal differences may also be important for understanding the insecticidal efficacy observed in Fig. 1 and Fig. 2.


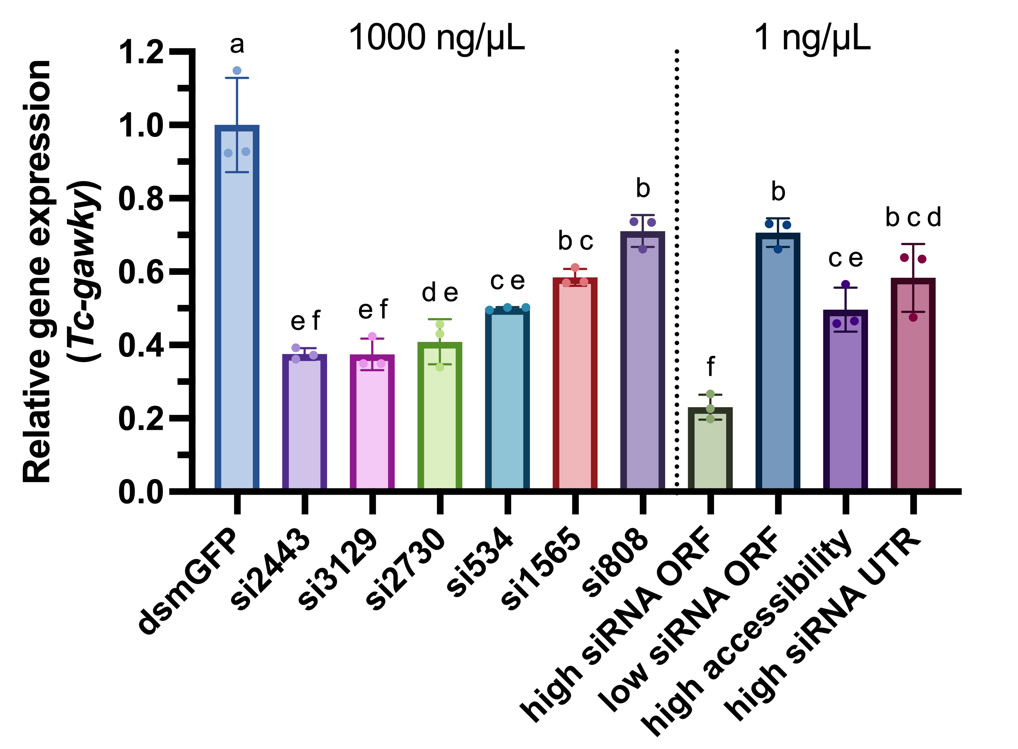


**Figure S3. Gene expression of *Tc-gawky* following single siRNA inserted or long complementary dsRNA injection.** The gene expression (mean ± SEM) of *Tc-gawky* was measured 3 days post injection of L5 larvae (3 biological replicates) through RT-qPCR. Total RNA from 5 whole larvae was extracted using Quick-RNA Tissue/Insect Kit (Zymo Research, Germany) and 100 ng RNA was used as input for RT-qPCR reactions with Luna® Universal One-Step RT-qPCR (NEB, Germany). Gene expression was normalized to the expression level in dsmGFP injected group (1000 ng/µL) using CFX Maestro Software (v1.0, Bio-Rad). One-way ANOVA followed by Tukey tests were performed. Groups significantly different from each other (P < 0.05) were annotated with different letters. Further details are provided in Additional File 2.


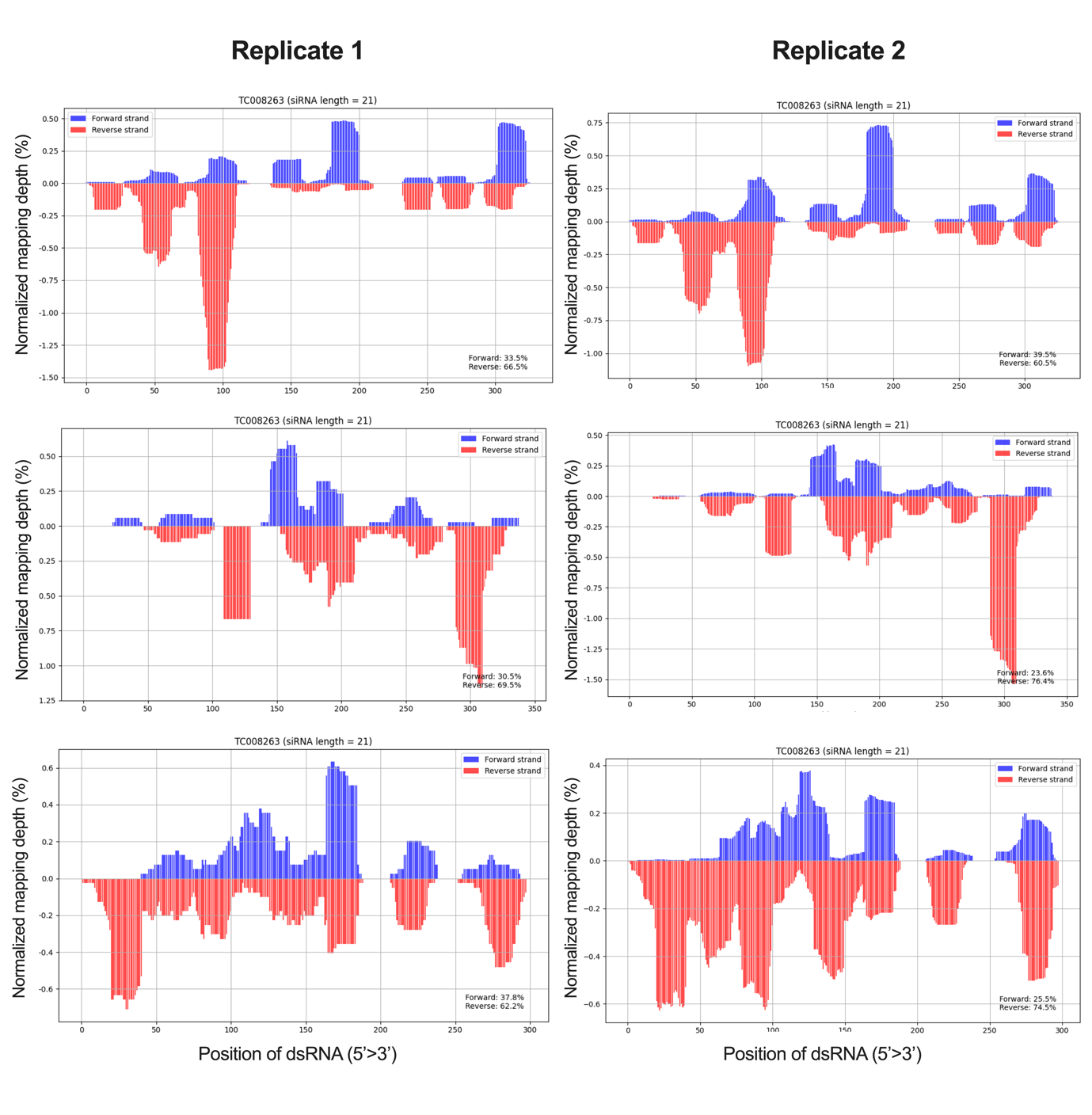


**Figure S4. Reproducibility of RISC-bound sRNA-seq experiments.** RISC-bound sRNA-seq was performed after the delivery of dsRNA targeting the essential gene Tc-*klp61F* (TC008263) to *T. castaneum* L5 larvae. The mapping depths obtained in two biological replications for each dsRNA are plotted separately (left and right plots). Same data are also used in Figure 3.
